# The Relationship between Food Safety Culture Maturity and Cost of Quality: An Empirical Pilot Study in the Food Industry

**DOI:** 10.3390/foods13040571

**Published:** 2024-02-14

**Authors:** Pauline Spagnoli, Lavinia Defalchidu, Peter Vlerick, Liesbeth Jacxsens

**Affiliations:** 1 Department of Food Technology, Safety, and Health, Faculty of Bioscience Engineering, Ghent University, Coupure Links 653, 9000 Ghent, Belgium; pauline.spagnoli@ugent.be (P.S.); defalchidulavinia@gmail.com (L.D.); 2 Department of Work, Organization, and Society, Faculty of Psychology and Educational Sciences, Ghent University, Henri Dunantlaan 2, 9000 Ghent, Belgium; peter.vlerick@ugent.be

**Keywords:** food safety culture, cost of quality, food processing industry

## Abstract

In the food industry, a mature food safety culture (FSC) is linked to better food safety performance. However, the relationship between FSC maturity and key economical performance indicators, such as cost allocation, remains unexplored. This research is the first pilot study to empirically explore the association between FSC maturity and cost of quality (CoQ). A CoQ survey was developed and pretested. CoQ data were collected through collaboration with company management. FSC maturity was assessed through a validated mixed-method assessment (diagnostic instrument, questionnaires, and interviews). A convenience sample of five food processing companies was assembled and subjected to FSC and CoQ assessment. Results revealed that monitoring CoQ is not yet standard practice in the food industry: three out of five companies were unable to specify all failure costs. For prevention and appraisal costs, results showed descriptively and statistically that when these costs are higher, FSC is more mature. Considering the theoretical context of the research (CoQ models and available literature), these results present the first empirical evidence to substantiate that FSC could replace product/service quality in CoQ models. Findings justify the push for a shift in perception, from considering FSC as a task on the list of resource demanding activities toward a narrative in which FSC contributes to financial health.

## 1. Introduction

The global food safety initiative defines food safety culture (FSC) as “shared values, beliefs and norms that affect mindset and behaviour toward food safety in, across and throughout an organization” [1] (p. 9). Nowadays, food safety culture is a highly relevant topic for the food industry. European legislation, [2], states that “food business operators shall establish, maintain and provide evidence of an appropriate food safety culture” (p. 6). Food safety culture is conceptualized by several researchers [3,4,5]. In this study, a specific FSC conceptual model [6] is followed, where food safety culture consists of three building blocks: the food safety management system (based on core control and assurance activities), the human–organizational, and the human–individual building block. Assessment reveals maturity of the prevailing food safety culture in a specific food processing organization. Maturity of FSC, or maturity of the dimensions that build FSC, is positioned on a scale with five-point maturity scales [7] (with stage one to five, respectively, representing the stage of doubt, react to, know of, predict, and internalize) and three-point maturity scales [8] (with stage one to three, respectively, representing a reactive, active, and proactive level of maturity) being most common. When a specific FSC dimension has a low maturity score, this dimension can be considered a gap [6]. A mature food safety culture is expected to result in higher levels of (microbiological) food safety of the products produced by the company. This relationship has been demonstrated by previous research [9], in which the link between food safety culture and *L. monocytogenes* control was investigated with the conclusion that a more mature food safety culture is associated with a lower *L. monocytogenes* contamination risk. Furthermore, another study [10] concluded that affiliated butcher shops achieve higher food safety output levels, because of a food safety management system and safety climate of higher maturity (which are both components of FSC). 

Monitoring the cost of quality (CoQ) is a method for managing the costs incurred to ensure that products/services meet quality standards, as well as the costs of goods/services that fail to meet quality standards. Studying cost of quality in processing firms helps to establish and maintain high quality levels while ensuring profitability [11]. A generally accepted definition of CoQ is that it is the cost of all efforts made by a company in order to provide a product that can meet the necessary requirements and the customers’ needs [12,13]. CoQ is relevant for many industries, going from construction [14] to the textile industry [15], healthcare [16], and the food industry [12,17]. Research on management accounting practices in the British food and drinks industry concludes that the calculation and reporting of cost of quality is likely to become more widespread [18]. Cost of quality is generally modeled against product/service quality, as between these a trade-off exists in which there is a point of minimal CoQ at a certain quality level [19], with the positioning of this point differing between models and theories (new versus old cost of quality model [20]).

Nowadays, food businesses are faced with a multitude of challenges and priorities, being, among others, food safety, sustainability, digitalization, and finding and keeping qualified workers. Because of a rising awareness of the importance of the topic, increasingly stringent private certification schemes, and legislation, companies will now have to put effort toward maturing their organizations’ FSC as well. This range of priorities underlines businesses’ limited (financial) means. Adding to all of this is the fact that companies simultaneously have to minimize customers’ costs because of increasingly complex and competitive business environments [21]. Within this current context, it is essential to increase available knowledge on the impact of the prominent trend of food safety culture on various aspects of food businesses. Research is already available on the link between FSC and food safety levels of products produced. Furthermore, it has been researched that leadership, centralization, and formalization significantly influence CoQ [22]. Put forth as the research question is whether and how FSC maturity in food businesses is related to cost allocation strategies (via the concept of cost of quality), which directly affects organizational performance [13,21]. If FSC influences CoQ within the company and vice versa, maturing food safety culture could contribute to economic gain [23]. Considering the common cost of quality models, the hypothesis arises that with an increasingly mature FSC, higher prevention and appraisal costs, lower failure costs, and lower overall quality costs come along. To the best of the authors’ knowledge, no prior empirical research has been performed on this topic. Industry members are urgently asking for evidence, to influence their company’s cost allocation plans and to convince business leaders and other stakeholders of the benefits a strong FSC brings. Linking the relatively novel concept of FSC (first publications around 2010, e.g., Ref. [24]) with the more established concept of CoQ (first publications on CoQ around 1970, e.g., Ref. [25]), through scientifically testing the stated hypothesis, is an important next step in FSC research as it positions FSC maturity and its added value within the bigger scope of a food processing organization. 

In this study, the PAF-model [26,27], or the prevention, appraisal, failure cost model, was selected to structure CoQ as it is the most recognized approach for quality costing [20] (the acronym PAF is formed with the first letter of each of the included costs). A cost of quality survey was developed based on available scientific research and pretested with six food producing companies. Next, the maturity of the prevailing food safety culture was assessed in a convenience sample of five other food processing companies through a validated food safety culture mixed-method assessment and gap analysis [6]. The same companies provided cost of quality data using the pretested survey. The relation between cost of quality and food safety culture was investigated descriptively and statistically (Pearson correlation and regression analysis). 

## 2. Materials and Methods

The research question of this study is whether and how FSC is related to cost allocation strategies, with the hypothesis that with an increasingly mature FSC, higher prevention and appraisal costs, lower failure costs, and lower overall quality costs come along (based on acknowledged CoQ models). In the five companies who joined the research, food safety culture maturity was assessed and cost of quality data were collected. Methods applied for assessing both concepts are described below.

### 2.1. Research Sample: Company Invitation Process and Description of Company Characteristics

To answer the research question of this study, company participation was crucial. Company criteria for study participation were the company must employ more than 10 people (micro companies were excluded, as per the definition by the European Commission [28], to ensure similar organizational structures), the company must preform a form of food processing activities (transformation, not solely distribution), and the company must have a production facility in Flanders, Belgium. To ensure validity of tools applied, the designed cost of quality questionnaire was pretested before application for the actual research. As explained in Section 2.2.2, in this pretest, quality managers evaluated the questionnaire’s comprehensibility, applicability, and availability of data asked. No CoQ data were retained from this pretest, as was made clear in the invitation email. For this pretest, six companies were approached via email and all answered positively, resulting in a 100% response rate. For the food safety culture assessment, no pretest was needed, as the tools applied are already part of a validated mixed-method methodology. For the next step, the actual investigation of the link between FSC maturity and CoQ, twenty companies were approached via an invitation email. This email contained a short explanation of the research, a guarantee of the confidential treatment of all data collected, and an invitation to join the study. Five out of these twenty approached companies decided to join the research on the link between CoQ and FSC, yielding a response rate of 25% (comparable response rates are found in similar studies, e.g., 21.3% [29]). It can be stated that it was difficult to convince the companies to participate in this part. This could be because of the sensitivity of the CoQ data, but also because most food companies are not monitoring their CoQ yet [13], implying that providing the data could have been considered as too time intensive by some companies. As study participation was voluntary, the assembled company sample was a convenience sample. Table 1 gives an overview of the characteristics of the six companies included in the pretest of the CoQ questionnaire, and the five companies included in the study on the link between CoQ and FSC. 

### 2.2. Cost of Quality Assessment

#### 2.2.1. Development of Questionnaire 

**Figure 1:**
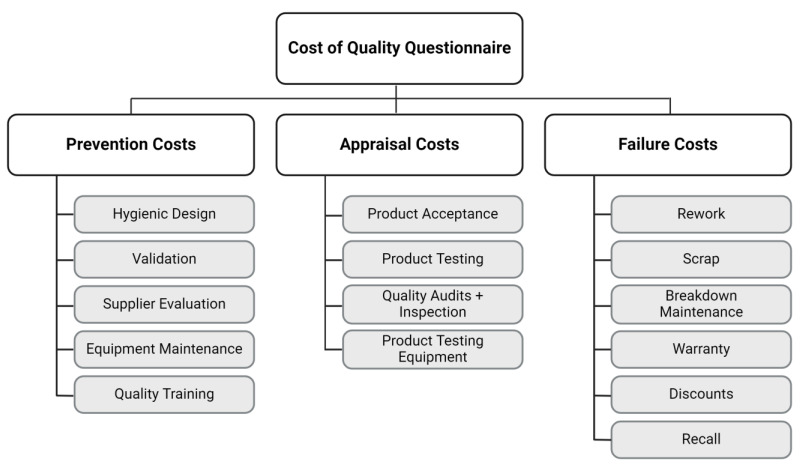
Structure of the applied cost of quality questionnaire, following the PAF-model (prevention–appraisal–failure), displaying the applied indicators per cost type [27].

To start data collection on CoQ, a CoQ survey was needed. A literature scan was conducted via Web of Science and Google Scholar to collect information on CoQ assessment in companies, with cost of quality as the search term. Previous work is available [17], which gives an overview of quality costing surveys worldwide up until 2018. Because limited literature is available on CoQ assessment in food companies specifically, the literature scan was broadened to CoQ assessment in general including all industries. Based on the inclusion criteria (i.e., available in English, access to full text, only articles and no proceeding papers, and only articles clearly describing a methodology to measure CoQ) and abstract reading, nine studies [12,13,17,21,29,30,31,32,33] were selected, analyzed for methodology and used for developing the questionnaire. The result was a CoQ assessment survey, divided into four sections, starting with an informed consent form. The first section is the introductory questions: “what is your company’s name?” (needed to link the FSC maturity analysis of each company to the CoQ data), “what is your company’s total annual sales (€)?” (added after the pretest), “do you measure cost of quality?” [17], “if yes, indicate the benefits of measuring CoQ” [12,30], and “if not, why not? Are there barriers or difficulties?” [12,17,30]. The following three sections asked for detailed information regarding each cost for the PAF-model, which are the prevention costs (five indicators), the appraisal costs (four indicators), and failure costs (six indicators), respectively (Figure 1). The PAF-model was elected as it is the most recognized approach for quality costing [20]. The indicators were selected from PAF-model literature [13,34,35,36,37], based on relevance for the sample (food industry) whilst staying as comprehensive as possible. The goal was not to obtain all details on the cost of quality in each company, but rather to collect data on these selected indicators to compare between companies in the sample. 

#### 2.2.2. Pretest of Questionnaire 

A pretest with practitioners was organized with six quality managers from Belgian food producing companies. This was performed in a workshop format, so all managers could give live feedback and discuss amongst each other. Characteristics of the pretest companies can be found in Table 1. The respondents were presented with a printed version of the questionnaire and were invited to read the survey with the aim of evaluating the questionnaire’s comprehensibility, applicability, and availability of data asked. No CoQ data were retained from this pretest, only comments regarding the survey. Based on this pretest, minor changes were made to the questionnaire as follows: The first change was asking the respondent to provide each cost in EUR/year and not in a percentage of total annual sales as this was deemed confusing. However, to be able to compare costs between different sizes of companies, a question concerning the company’s total annual sales was added in the first section (so percentages of sales could be calculated by the researchers after completion of the questionnaire). Calculating the percentage of each cost of the total annual sales is widely performed in CoQ literature [29,35], as it facilitates comparison. Furthermore, as it was not always clear for practitioners what exactly to include in the costs, a short explanation for each cost was added per question, together with a ‘formula’ stating what exact costs to include to standardize data collected (e.g., for the prevention cost ‘supplier evaluation costs’, the question becomes “can you give us an estimation of your yearly supplier evaluation costs? (i.e., the process of assessing and approving potential suppliers through quantitative and qualitative assessments) = salary (time) of the employee (e.g., quality manager) spent to evaluate suppliers + transportation costs to do supplier audits/year”). The revised and definitive version of the developed cost of quality survey is presented as Supplementary Materials.

#### 2.2.3. Cost of Quality Data Collection and Analysis

After the pretest of the CoQ questionnaire and implementation of slight alterations as described in Section 2.2.2, the quality managers of the five participating companies (different companies than those included in the pretest of the questionnaire) received the CoQ questionnaire digitally with an invitation to an online session to fill in the questionnaire guided by the researchers. The quality managers were the main contact persons, but worked together with various departments, like the financial department, to collect the relevant data needed to complete the questionnaire (which is in line with previous research [29]). All companies chose to fill in the questionnaire independently, so not in a guided session format, as they estimated the data collection had to be performed in a period of several days, collecting data from several departments, demanding multiple internal conversations or meetings. If questions came up during the data entry process, researchers were continuously available via email. Once finished, companies returned the questionnaire to the researchers in a Word or pdf file format. 

Data were transferred from the files provided by the companies to one integrative Excel file. Researchers anonymized all data. The CoQ data gathered with the questionnaire are expressed in euros and are estimations made by the companies about their own situation, independently from each other. These costs provided in euros were transformed to the percentage of the total annual sales of the company, to facilitate comparison [29,35]. The percentage of the annual sales for each category of the PAF-model (prevention, appraisal, and failure costs) was calculated by finding the sum of the indicators (as is generally accepted and performed, e.g., in Ref. [14]).

### 2.3. Food Safety Culture Maturity Assessment 

FSC consists of three building blocks: the food safety management system (FSMS), the human–organizational, and the human–individual building block [6]. The FSC assessment applied in this research is the assessment proposed and validated in Refs. [6,38]. In each of the five companies, the methodology described below was applied to determine the maturity and gaps in maturity of the prevailing FSC. 

The FSMS (core control and assurance activities) was assessed in each company with the food safety management system diagnostic instrument (FSMS-DI). In line with previous research, the diagnosis was made through a standardized in-person interview with the quality manager of each food company, providing insights concerning the riskiness of the context [39], the level of control activities [40], and the level of assurance activities [41]. Maturity in this tool is scaled as one to three, respectively, indicating a low, medium, and high level of the control/assurance activities. 

The human–organizational building block was assessed using the food safety climate tool/questionnaire [6,42]. Employees’ perceptions (all employees, including managers, operators, technical staff, etc.) on five dimensions (leadership, communication, commitment, resources, and risk awareness) were evaluated with a self-assessment survey based on a five-point Likert scale (from totally disagree to totally agree) via the food safety climate tool. Most employees could fill in the questionnaire independently. However, where needed, researchers organized fill-in sessions for those employees needing extra help interpreting the questions, e.g., due to language barriers. The questionnaire was filled in voluntarily and anonymously to ensure the privacy of the participants. Next, the human–organizational building block was also assessed with the card-aided management interview [6], specifically its dimensions concerning adaptability, consistency, beliefs and values and vision, mission, and strategy, which were evaluated by all the managers in each company on a scale of reactive (maturity level 1) to proactive (maturity level 3). This was performed, per company, in a live interview with all managers of the company together to facilitate discission on maturity as recommended [6]. 

To assess the human–individual building block, all employees individually filled out a self-assessment questionnaire containing indicators of the dimensions of compliance, participation, motivation, knowledge, stress, and burnout [3,43,44,45]. These survey questions, as selected from the literature, have 5- and 7-point Likert answer scales. In practice, these indicators were presented to employees together with the food safety climate tool. The questionnaire was filled out voluntarily and anonymously to ensure the privacy of the participants. Ultimately, 90%, 77%, 77%, 100%, and 58% of employees filled in the combined questionnaires in company 1 to 5, respectively. 

To be able to make a conclusion of overall FSC maturity of each company, data of the mixed-method assessment were integrated (combined) [6]. This entailed providing one maturity score per FSC dimension through combining responses from respondents and by rescaling all scores on a maturity scale of one to three (by calculating the mode for the management interview and assigned scores allocated based on the mean of responses for the FSMS-DI and questionnaires) [6]. When a dimension had a maturity score lower than 2_3 on the scale of 1 to 3, this dimension was considered a gap, or a dimension that is immature [6]. 

### 2.4. Data Analysis of the Link between Cost of Quality and Food Safety Culture 

The relation between cost of quality and food safety culture was investigated descriptively and statistically. A descriptive analysis was performed by making a scatter plot of the FSC and CoQ data, so trends can be visually observed. For the statistical analysis, Pearson correlation and regression analyses were performed (0.05 significance level), using the percentages of total annual sales each type of cost (prevention/appraisal/failure) accounted for (calculated by finding the sum of the indicators) and the number of food safety culture gaps (i.e., underdeveloped dimensions). IBM SPSS Statistics version 28 (Chicago, IL, USA) was used to perform the analysis.

## 3. Results and Discussion

### 3.1. Cost of Quality in Study Sample

This section focuses solely on the results of the cost of quality assessment. Section 3.2. will discuss food safety culture maturity in the sample. Section 3.3, in its turn, will investigate the FSC-CoQ relationship. 

#### 3.1.1. Frequency of Monitoring, Barriers, and Benefits 

In the first section of the CoQ questionnaire, respondents were asked if their company already measures CoQ and why/why not (Table 2). Two (company 1 and company 3) out of five companies say they do not monitor CoQ yet. When asked why, both selected the following: ‘complexity in implementing a cost of quality system: no guidelines’, ‘lack of knowledge of CoQ principles’, and ‘difficulties in collecting data’. Previous research [17] investigated the barriers for CoQ implementation and showed that the primary difficulty is the complexity in implementing a cost of quality system. Company 1 additionally selected ‘insufficient budget’ and ‘lack of adequate accounting and computer systems necessary to track CoQ’ as barriers. Previous studies have shown that CoQ remains generally unmonitored within the food industry and beyond [13,33]. As the sample in this study is a convenience sample, it is expected that monitoring CoQ served as a prerequisite to join the research. 

In contrast, companies that do measure CoQ (three out of five companies in the sample) reveal to indeed have found several benefits, such as ‘product/service quality improvement’, ‘increase in customer and employee satisfaction’, ‘decrease in customer complaints’, ‘elimination of all forms of waste’, ‘increase in profit’, ‘increase in company competitiveness’, and so on (Table 2). This is confirmed by previous research, e.g., by the author of [46], who studied CoQ in five hundred industrial enterprises in Turkey. In this work, it was demonstrated that implementing a CoQ system leads to, among other effects, a reduction in customer complaints, a reduction in rework, scrap, and warranty expenditures, and an increase in sale volume. 

#### 3.1.2. Cost of Quality Data 

This section purely discusses CoQ in the sample. Table 3 provides the overview of this cost of quality data in the five participating companies. The table displays the costs per category of the PAF-model, per indicator of each category as provided by the companies. The costs are expressed as the percentage of the cost of the total annual sales of the company. Costs of the individual indicators are summed to provide an indication of the total prevention, appraisal, and failure costs, respectively. 

The prevention costs range from 3.87% of the total annual sales of company 1 to 0.86% of the total annual sales of company 5 (Table 3). These results are in line with findings from similar research, where an amount of 2.0% and 4.0% of total annual sales was calculated for prevention costs in a hotel restaurant [35]. Company 2 indicated that it had an exceptional year in terms of investments in hygienic design, which is not representative of other years. This was discussed with the company concerned, whereafter it was concluded that for normal years, the hygienic design costs are already included in the validation costs. A similar situation was encountered in company 4, as the costs provided for the hygienic design indicator included all validation costs. It was not possible for this company to differentiate costs to a more detailed degree. However, as the sum of the indicators is used per company in further analyses, this does not influence results.

Appraisal costs range from 1.25% (of the total annual sales) in company 1 to 0.21% in company 5. Compared to previous studies, where an amount of 2.0% and 4.0% of total annual sales was calculated for appraisal costs in a hotel restaurant [35], these results are quite low. Of course, a food processing company and a hotel restaurant are very different environments, which could explain differing results. Company 4 was not able to split data of the indicators of product acceptance costs (i.e., the verification of raw materials to check if they are usable for their intended purpose) and product testing costs (i.e., process of measuring the properties or performance of finished products). 

Concerning the failure costs, a lot of data are missing (Table 3), as companies were unable to provide these data. This is a problem encountered before by other researchers (e.g., Ref. [13]). Maybe some departments within companies hesitated to provide the data, because they are concerned about the company’s reputation or are worried this could influence their performance evaluation [47]. Due to the missing data, discussion on the total failure costs (sum of all types of failure costs, as performed for prevention and appraisal costs) was avoided. Instead, it is more interesting here to zoom in on the individual failure cost types. There is only one indicator that all companies provided, which is the recall costs. All companies said they did not have any recall costs in recent years, except for company 5, who estimated a 0.04% of total annual sales in recall costs. Rework costs range between 0.00% for company 3 and 0.16% for company 2. For several types of the included costs in this study, Crosby has proposed limits in his book *Quality is Free*, which can be used to compare the collected data with. Crosby gives 0.25% of sales as an allowed amount for rework costs [48], to which all companies comply. Crosby proposed the same amount, 0.25% of sales, as an allowed amount for scrap. All companies spend less than 0.25% on scrap, except for company 1. Company 1, which is the company with the lowest FSC maturity, spends 3.89% of sales on scrap, which is far above the limit proposed by Crosby [48]. For warranty costs, 0.2% of sales is allowable. Again, only company 1 exceeds this limit with 0.39% of warranty costs. 

Two out of five companies were able to specify all costs. So, for these companies, the total cost of quality can be calculated by finding the sum of the prevention, appraisal, and failure costs. For company 2, the total cost of quality is 3.42% of total annual sales. For company 3, the total cost of quality equals 1.56%. Crosby gives an estimated reported and actual amount of cost of quality for each of the five stages in his quality management maturity grid. Stages one to five have, respectively, an unknown percentage, 3%, 8%, 6.5%, and 2.5% of reported cost of quality as a percentage of sales. The actual percentage for the five stages is 20%, 18%, 12%, 8%, and 2.5% [48]. Considering the relatively mature FSC of both companies (four gaps, see Section 3.2), and the description of the quality management maturity stages by Crosby, it is estimated that both companies are close to the highest maturity stage in Crosby’s quality management maturity grid. In this most mature stage, named ‘certainty’, cost of quality consists almost entirely of compensation for employees of the quality department and the costs of proofing tests [48].

### 3.2. Food Safety Culture Maturity and Gap Analysis

Table 3 provides an overview of both food safety culture maturity (number and identity of identified gaps) and cost of quality data in the five participating companies. The CoQ data are addressed in the previous paragraphs (Section 3.1.2), while this section focuses on the maturity of food safety culture in the companies. Section 3.3 will in turn discuss the CoQ-FSC relation. The first row of Table 3 displays this FSC maturity, specifying which dimensions were underdeveloped or a gap. Companies 1 to 5 have, respectively, 3, 4, 4, 5, and 8 gaps in their prevailing FSC maturity. Company 1 and 2 only have gaps in the human–organizational building block, whilst companies 3 to 5 have gaps in both the FSMS and the human–organizational building block. All companies have a gap for the dimensions ‘adaptability’, ‘consistency’, and ‘mission & vision’, which are very common FSC gaps [38]. For the company with only three gaps, these dimensions are the only underdeveloped ones. For the other companies, additional dimensions are underdeveloped (i.e., gaps), as displayed in Table 3.

### 3.3. Relationship between Food Safety Culture Maturity and Cost of Quality

The main goal of this research is to investigate if a relationship or association can be observed between FSC maturity and CoQ in food processing organizations. For each company (five in total), the percentage of sales for each type of cost (prevention and appraisal cost) is used in the analysis, which is the sum of the indicators, together with the number of gaps in FSC maturity. The sum of prevention and appraisal costs is also used in the analyses. Failure costs are not included because of the inability of the companies to provide these data (Table 3). 

#### 3.3.1. Descriptive Analysis and Interpretation of the Relation

**Figure 2:**
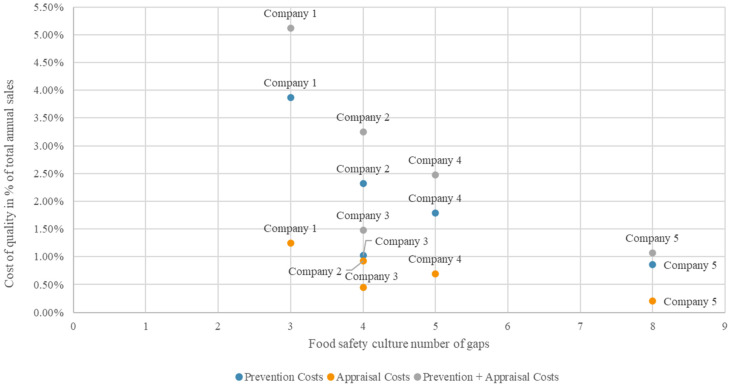
Relation between food safety culture (FSC) maturity (expressed as the number of gaps identified through the maturity assessment) and cost of quality (following the PAF-model and expressed as the percentage of total annual sales). Five companies are included in the sample, with, respectively, 3, 4, 4, 5, and 8 FSC gaps. Prevention and appraisal costs are included in the figure, as well as the sum of both.

Figure 2 provides a scatter plot on which the relation between food safety culture (FSC) maturity (expressed as the number of gaps identified through the maturity assessment) and cost of quality (following the PAF-model) becomes visible. Five companies are included in the sample, with 3, 4, 4, 5, and 8 FSC gaps. Prevention and appraisal costs are included in the figure, as well as the sum of both. Based on Figure 2, results clearly show a positive relationship between FSC maturity (i.e., the number of gaps, in each of the five companies) and both prevention and appraisal costs (i.e., the percentage of annual sales the costs account for, in each of the five companies). As FSC is more mature when there are less gaps, the relation between the number of gaps and the prevention and appraisal costs is negative. So, the more mature the FSC, or the less gaps present, the more expenses are made for prevention and appraisal. Interestingly, there are two companies with four gaps, but with various levels of prevention and appraisal costs. Company 2 has gaps in the dimensions of adaptability, consistency, beliefs and values, and mission and vision, whilst in company 3, the dimensions control activities, adaptability, consistency, and mission and vision are a gap (Table 3). So, the difference in FSC maturity is that company 2 has a gap for the dimension beliefs and values, whilst company 3 has a gap for the dimension of control activities as part of the food safety management system. Company 2 has higher prevention (2.32%) and appraisal costs (0.93%), compared to company 3 (1.03% prevention and 0.45% appraisal costs). From this, it could be speculated that it is more costly to improve control activities compared to beliefs and values within the organization. This makes sense, as improving control activities more often means technical investments, e.g., in infrastructure or technology, whilst improving beliefs and values means working with people, e.g., by organizing a safety priority setting activity [49] or by encouraging employees to want to be part of the social norm [50]. Alternatively, it could be argued that company 2 has made a lower return on investment, as the number of gaps is the same and company 2 has higher costs. 

#### 3.3.2. Statistical Analysis and Interpretation of the Relation

The relationship between the two concepts of FSC and CoQ was statistically tested by calculating the Pearson correlation coefficient. This analysis returned a correlation coefficient of −0.701 and a *p*-value (2-tailed) of 0.188 for the prevention costs, and a correlation coefficient of −0.815 and a *p*-value (2-tailed) of 0.093 for the appraisal costs. Performing this analysis with only five data points has little power, which could explain the *p*-value of more than 0.05. However, both negative correlation coefficients confirm the trend depicted in Figure 2 and suggest a positive relationship between the maturity of companies’ food safety culture and both prevention and appraisal costs (negative coefficients as the FSC is more mature when there are less gaps). When the same analysis is made for the combination of appraisal and prevention costs (sum of appraisal and prevention costs’ percentage of annual sales), comparable results are observed: a positive relationship is visible, with a correlation coefficient of −0.734 and a *p*-value (2-tailed) of 0.158. To further explore the relationship between CoQ and FSC, a linear regression analysis was performed based on the scatter plot in Figure 2. First, the variable “number of gaps” was added as the independent variable, which could influence the dependent variable of “prevention costs”. This yielded an R square value of 0.491 and a statistical significance of the regression model of 0.188. The regression equation is the following: prevention costs = 4.094 − 0.442 × (FSC Gaps). For the analysis of FSC vs. appraisal costs, results show an R square value of 0.665 and a statistical significance of the regression model of 0.093. The regression equation is the following: appraisal costs = 1.531 − 0.172 × (FSC Gaps). For the analysis of FSC vs. prevention + appraisal costs, results show an R square value of 0.538 and a statistical significance of the regression model of 0.158. The regression equation is the following: prevention + appraisal costs = 5.625 – 0.614 × (FSC Gaps).

In this cross-sectional study, data on both variables (food safety culture and cost of quality) were collected in the same timeframe and compared between cases. The revealed positive relationship between FSC and both prevention and appraisal costs could suggest that higher prevention and appraisal costs foster a more mature food safety culture. Alternatively, results might reflect that maturing food safety culture fuels higher costs in prevention and appraisal. Lastly, the described relationship could also be reciprocal or bidirectional. However, as study variables were measured at a similar time point instead of measuring them over time through using a longitudinal research design, causal interpretations should be avoided. Future research might study, in a case study, whether CoQ shifts over time due to a FSC intervention (e.g., educational actions as described in Ref. [51]) through comparing CoQ pre vs. post intervention. Furthermore, one might discuss the nature or form of the discovered relationship. It might be that the relationship between the cost of qualities (prevention, appraisal, or failure costs) and FSC is not linear (as assumed in the linear regression analysis based on the scatter plot). Maybe more investments are needed to go from three to two gaps and then from eight to seven gaps in food safety maturity, for example. A previous study [23] aligns five FSC maturity stages to five percentages of sales of the cost of poor quality, which shows a roughly linear relationship when converted into a scatter plot. 

#### 3.3.3. Discussion on the Attributes of the Relation and Interpretation of Findings 

Results might be influenced by unmeasured company characteristics, like the product produced or the production process. These characteristics might influence the cost of quality, the food safety culture maturity in the company, and the relationship between both. Research concerning the influence of company characteristics on cost of quality itself, going beyond only studying the level of implementation of a cost of quality system or the level of monitoring (e.g., Ref. [52]), is extremely limited. FSC maturity could itself be a company characteristic influencing CoQ. Previous work [53] states that organizational culture is one of the reasons why organizations do not collect quality costs. The influence of factors like company size is mitigated by comparing and calculating CoQ as the percentage of the total annual sales. Certification could also influence CoQ or cost efficiency [54]. It was demonstrated [55] that the quality management maturity in food and beverage enterprises is related to the present system for quality costing and the focus of this system. The country could also be an influencing factor, as it was shown [56] that FSC is dependent on the company’s geographical location, because of food safety governance and national values. The cost of quality could also be influenced by location. A Web of Science region analysis (2023) of “cost of quality” results demonstrates that 39% of results originate from North America, indicating a much bigger focus on the topic in this region compared to the rest of the world. International research is recommended to investigate possible moderators and/or boundary conditions influencing the relationship between cost of quality and food safety culture maturity.

In this research, cost of quality entails costs incurred for the food safety management system (prevention and appraisal costs) and costs caused by products or equipment that are below par (failure costs), as in traditional cost of quality constructs. Concerning the prevention and appraisal costs, it was shown that when these are higher, the overall FSC is more mature or less gaps prevail within a specific company. Translated to the FSC conceptual model [6], this means that investments in the prevention and appraisal aspects of the food safety management system (first building block of FSC) might foster the improvement in other building blocks as well (i.e., human–organizational and human–individual). But what about costs relating to the other building blocks in food safety culture? Some studies include “social non-quality (work accident, absenteeism, demotivation, presenteeism, etc.)” [17] as a cost of non-quality in cost of quality assessment [17]. This raises the question that maybe, within the framework of FSC research, other costs could also be linked to FSC maturity, therefore possibly creating even more motivation or return on investment for companies to improve their FSC. For example, maybe maturing FSC causes social non-quality costs to become smaller, like demotivation, or maybe an immature FSC causes higher psychological costs (e.g., enhanced rumination, turnover intention, and burnout; lowered mental health among staff)? The latter can be demonstrated by, e.g., the concept of whistleblowers. It is known that whistleblowing can cause psychological harm to the whistleblower [57]. In an organization with a strong FSC, whistleblowing is expected to be much less necessary but therefore taking away pressure from the whistleblowers. 

Despite the discussion above, results should be interpreted within the theoretical context of the research. Following the new cost of quality model, which is more in congruence with empirical findings [58], it is accepted and demonstrated in CoQ theory and research that when the quality of the products increases, prevention and appraisal costs increase, failure costs decrease, and the total CoQ decreases (with a minimum at the highest quality level). A study [59] found Pearson correlation coefficients between 0.549 and 0.620 for the relationship between quality and prevention + appraisal costs, with all *p*-values < 0.005. This empirical study, in its turn, revealed that higher levels of food safety culture maturity are also associated with higher prevention costs and appraisal costs and the sum of both. Furthermore, academics and practitioners agree that a mature FSC (consisting of the food safety management system, food safety climate, and other human dimensions on the organizational and individual level) and high food safety output levels go hand in hand [9,10], therefore linking product quality and FSC. Returning to the hypothesis formulated in the Introduction, the discovered positive association between FSC and prevention/appraisal costs is the first empirical proof that FSC could replace product/service quality in the CoQ models. In addition, Ref. [60] found that failure costs are negatively affected by a rational organizational culture. When combined, this suggests that a more mature FSC is not only linked to higher appraisal and prevention costs, but also to lower failure costs, lower overall quality costs, and better organizational performance for the company [12,21]. Previous research further solidifies this. It was discovered [61] that four cultural traits or dimensions (involvement, consistency, adaptability, and mission, as assessed in the FSC assessment in this study) showed a significant positive association with subjective and objective measures of organizational effectives, like return on assets and sales growth. In addition, causality was demonstrated in a longitudinal study [62]: positive organizational culture traits lead to greater customer satisfaction in later years, positively influencing performance. Findings from this study have major managerial implications as they deliver the first empirical evidence to justify the push for a shift in perception, from considering FSC as an extra task on the list of resource-demanding activities toward a narrative in which maturing FSC is a potential pathway toward increased financial health and stability of food processing companies [23].

## 4. Conclusions

This paper is the first to empirically investigate the relationship between FSC and CoQ. The first result was that collecting and monitoring CoQ data is not yet standard practice in the food industry. This was especially true for failure costs, as three out of five companies were unable to specify all types of failure costs. Next to this, available CoQ data within the food industry are extremely scarce, which makes the presented data in this study an important addition to the research field. 

Results from the FSC-CoQ relation analysis revealed a trend, namely a positive relationship between, on the one hand, prevention costs, appraisal costs, and prevention plus appraisal costs and, on the other hand, food safety culture maturity. In other words, going back to the FSC conceptual model employed, this research provided evidence that a higher overall maturity of the dimensions that build FSC, i.e., control and assurance activities, leadership, communication, resources, commitment, risk awareness, consistency, adaptability, beliefs and values, mission and vision and strategy, knowledge, participation, motivation, compliance, and psychosocial well-being, is associated with more financial investments in prevention and appraisal quality costs. Returning to the hypothesis formulated in the Introduction and considering the theoretical context of the research (i.e., CoQ models), the discovered positive association between FSC and prevention/appraisal costs is the first empirical proof that FSC could replace product/service quality in the CoQ models. This could mean, as substantiated by the most recent CoQ models, that a more mature FSC is not only linked to higher appraisal and prevention costs, but also to lower failure costs, lower overall quality costs, and a better organizational performance for the food processing company. Study findings extend available knowledge on FSC and its correlates, and provide leverage to convince business leaders in the food industry to allocate funds more deliberately, taking food safety and FSC into account when investing. The strategic importance of food safety culture is reaffirmed and underlined. Because of the limited sample size, a future large-scale empirical, preferably longitudinal, study with an in-depth data analysis is recommended to further solidify findings.

## Figures and Tables

**Table 1:** Company characteristics of companies included in the pretest of the cost of quality questionnaire (company A–F) and case companies included in the research on the link between food safety culture maturity and cost of quality (company 1–5). Companies were asked if their company is part of a group of companies; if yes, the following was asked: how many companies are in this group, if their company is a family business, how many full-time employees are working in the company, how many food safety trainings are organized per year, if their company produces animal- or plant-based products, if the products produced have a relatively long (e.g., frozen products) or short shelf life (e.g., fresh soups), if the company exports internationally, if the company focuses on B2B or B2C sales, if products produced are private label or A-brand, and what certificates the company holds.

Company	Group	Sites in Group	Family Business	FTEs	Yearly FS Trainings	Place in the Chain	Plant/ Animal- Based	Shelf Life	International Export (Outside EU)	B2B/B2C	A-Brand, Private Label	Certificates
Cost of quality questionnaire pretest												
A	No		Yes	42	>4	Trans/distr	Mix	Long	No	Both	Both	/
B	No		Yes	17	>4	Trans/distr	Plant	Short	Yes	B2B	Private label	IFS
C	No		No	140	>4	Trans	Plant	Long	Yes	B2B	A-brand	FSSC 22000,
D	Yes	>4	No	330	1	Trans/distr	Animal	Short	No	Both	Private label	IFS, SCS
E	No		Yes	60	2	Trans	Plant	Long	Yes	B2B	Both	BRC, IFS, SCS
F	No		Yes	30	1	Trans	Plant	Short	No	Both	Both	IFS
Case study on the relation between food safety culture maturity and cost of quality												
1	No		No	30	>4	Trans	Plant	Long	Yes	B2B	Private label	IFS
2	No		Yes	48	1	Trans	Plant	Long	Yes	Mix	Mix	BRC, IFS, SCS
3	No		No	95	>4	Trans	Mix	Short	No	B2B	A-brand	IFS, SCS
4	No		Yes	31	1	Trans	Animal	Long	Yes	Mix	Mix	IFS, SCS
5	Yes	>4	No	250	1	Trans	Mix	Short	No	B2B	Mix	IFS, SCS

FTEs: full-time employees; FS: food safety; trans: transformation of food products; distr: distribution of food products; B2B: business to business; SCS: self-checking system as required from the Belgian food safety authority; IFS: International Featured Standard; BRC: British Retail Consortium; FSSC 22000: Food Safety System Certification Scheme 22000; / means the company has no certificates.

**Table 2:** Overview of companies’ answers on the introductory questions of the cost of quality (CoQ) questionnaire, concerning their assessment of their own CoQ, and the benefits or difficulties that come along with doing so.

Company	Do You Already Measure CoQ?	If ‘Yes’, What Are The Benefits?/If ‘No’, Why Not? Are There Barriers or Difficulties?
1	No	Complexity in implementing a cost of quality system: no guidelines Lack of knowledge of CoQ principles Difficulties in collecting data Insufficient budget Lack of adequate accounting and computer systems necessary to track CoQ
2	Yes	Achievement of significant cost reductions Decrease in customer complaints Elimination of all forms of waste
3	No	Complexity in implementing a cost of quality system: no guidelines Lack of knowledge of CoQ principles Difficulties in collecting data
4	Yes	Increase in profit Increase in company competitiveness
5	Yes	Product/service quality improvement Increase in customer and employee satisfaction Decrease in customer complaints Elimination of all forms of waste

**Table 3:** Overview of food safety culture maturity expressed as number of gaps identified based on data from the maturity assessment, according to the method in Ref. [6], and cost of quality data in the five participating companies. The first row displays the FSC maturity, specifying which dimensions were still underdeveloped or a gap. The next rows clarify the costs per category of the PAF-model, per indicator of each category, as provided by the companies. The costs are expressed as the percentage of the cost of the total annual sales of the company.

	Company 1	Company 2	Company 3	Company 4	Company 5
Food safety culture maturity or number of gaps	Three gaps: adaptability, consistency, mission and vision	Four gaps: adaptability, consistency, beliefs and values, mission and vision	Four gaps: control activities, adaptability, consistency, mission and vision	Five gaps: control and assurance activities, adaptability, consistency, mission and vision	Eight gaps: control and assurance activities, commitment, resources, adaptability, consistency, beliefs and values, mission and vision
Prevention costs					
Hygienic design	2.22%	(=included in validation)	0.06%	1.20%	0.02%
Validation	0.07%	0.035%	0.15%	(=included in hygienic design)	0.04%
Supplier evaluation	0.18%	0.009%	0.02	0.02%	0.005%
Equipment maintenance	1.11%	2.25%	0.75%	0.55%	0.78%
Quality training	0.28%	0.025%	0.05	0.015%	0.01%
Total prevention costs	3.87%	2.32%	1.03%	1.79%	0.86%
Appraisal costs					
Product acceptance	0.08%	0.265%	0.06%	0.60%	0.04%
Product testing	0.67%	0.45%	0.10%	(=included in product acceptance)	0.11%
Quality audits	0.33%	0.20%	0.28%	0.08%	0.05%
Product testing equipment	0.17%	0.019%	0.01%	0.01%	0.014%
Total appraisal costs	1.25%	0.93%	0.45%	0.69%	0.21%
Failure costs					
Rework	Data not available	0.16%	0.00%	0.06%	Data not available
Scrap	3.89%	0.01%	0.06%	Data not available	0.05%
Breakdown maintenance	Data not available	0.00%	0.00%	Data not available	Data not available
Warranty	0.39%	0.002%	0.02%	Data not available	Data not available
Discounts	0.00%	0.00%	0.00%	0.007%	Data not available
Recall	0.00%	0.00%	0.00%	0.00%	0.04%
Total failure costs (sum of available data)	4.28%	0.17%	0.08%	0.07%	0.09%

## Data Availability

Data is contained within the article or Supplementary Materials.

## Supplementary Materials

The following supporting information can be downloaded at: https://www.mdpi.com/article/10.3390/foods13040571/s1, Questionnaire cost of quality.
