# Supplementary material for: The Relationship between Food Safety Culture Maturity and Cost of Quality: An Empirical Pilot Study in the Food Industry
Source: Foods. 2024 Feb 14;13(4):571. doi: 10.3390/foods13040571 (PMC10887550; doi:10.3390/foods13040571)
Supplement: Supplementary file 1 [file foods-13-00571-s001.zip › foods-2864317-supplementary.pdf]

QUESTIONNAIRE COST OF QUALITY

Dear participant,

Below you will find a questionnaire for the Q-DNA project, carried out by Ghent University. The Q-DNA project is about food safety culture, in which the human aspect in food safety is important. The goal of this questionnaire is to collect data on cost of quality in the food industry. This data will be used to investigate the link between CoQ and food safety culture. **Data is completely confidential**, so try to answer as honestly as possible. We ask you to read the questions carefully and provide the most appropriate answer for your company.

Consent form

We ask you to first read this form carefully and indicate what you agree with or do not agree with the following statements.

Hereby I declare that:

- My participation is completely **voluntarily** and that I know that I can **stop my participation at any given moment;**
- I give permission to **anonymously** use the collected **results,** also for other researchers;
- I am aware that not participating or stopping my participation **will never cause negative consequences** for me;
- I know that I can get a **summary** of the study on request after the study has been completed and the results are all collected;

☐ I declare that I have received sufficient information about this research and the opportunity to ask additional questions.

☐ I declare that I agree with this consent form.

© Ghent University

SECTION 1

1. What is the name of your company?
   (So we can link FSC and COQ)

   ____

2. What are your total annual sales (in €)?

   ____

3. Do you already measure cost of quality?
   ◯ Yes
   ◯ No

   ▪ **If yes:**
     • Which are the benefits?
     ◯ Increase in profit
     ◯ Increase in sales volume
     ◯ Increase in company competitiveness
     ◯ Product/service quality improvement
     ◯ Achievement of significant cost reductions
     ◯ Increase in customer and employee satisfaction
     ◯ Decrease in customer complaints
     ◯ Elimination of all forms of waste
     ◯ Others: _______

   ▪ **If no**:
     • Why not? Are there barriers or difficulties?
     ◯ Complexity in implementing a Cost of Quality System: no guidelines
     ◯ Lack of knowledge of CoQ principles
     ◯ Difficulties in collecting data
     ◯ Insufficient budget
     ◯ No interest on implementing a Cost of Quality System
     ◯ Lack of adequate accounting and computer systems necessary to track CoQ
     ◯ Others: _______

© Ghent University

SECTION 2

- **Prevention costs:** The prevention costs are the costs of all activities undertaken to prevent defects and problems, in order to achieve good quality.

1. Can you give us an estimation of your yearly costs for **hygienic design** and development of equipment?
   = cost of personalized hygienic design + cost of tests + salary (time) of employees + cost of external experts

   €____

2. Can you give us an estimation of your yearly **validation** costs (i.e. checking in advance the effectiveness of the technological and managerial measures aimed at controlling food safety, e.g. process validation, validation of preventive measures (e.g. validation of hygienic design, personal hygiene, raw material control, etc.), validation of CCP monitoring)
   = analysis/test cost + salary (time) of employees + cost of external experts

   €____

3. Can you give us an estimation of your yearly **supplier evaluation** costs? (i.e. the process of assessing and approving potential suppliers through quantitative and qualitative assessments)
   = salary (time) QM spend to evaluate suppliers + transportation costs to do supplier audits /year

   €____

4. Can you give us an estimation of your yearly **equipment maintenance** costs? (i.e. any process used to keep a business's equipment in reliable working order)
   = calibration costs + maintenance costs /year

   €____

5. Can you give us an estimation of your yearly **quality training** costs? (i.e. costs incurred to upgrade the technological skills of employees)
   = time spend of internal employee to prepare training + consultant/costs of external trainer + costs training materials (online platforms, programs, …) + time (salary) of employees spend following training /year)

   €____

© Ghent University

SECTION 3

- **Appraisal costs:** The appraisal costs are associated with the evaluation of purchased materials, processes, intermediate and final products, and services to assure conformance with the specified requirements.

1. Can you give us an estimation of your yearly **product acceptance** costs? (i.e. the verification of raw materials to check if they are usable for their intended purpose)
   = analysis cost + salary (time) of employees + product loss /year

   €____

2. Can you give us an estimation of your yearly **product testing** (labor and material) costs? (i.e. process of measuring the properties or performance of finished products)
   = analysis cost + salary of employees + product loss /year

   €____

3. Can you give us an estimation of your yearly costs of **quality audits and inspection** of production (i.e. verification of human, equipment and method related performance).
   = analysis cost + salary (time) of employees + cost of external experts

   €____

4. Can you give us an estimation of your yearly **product testing equipment** costs?
   (i.e. buying the equipment for analysis in house)
   = price of equipment for product testing

   €____

© Ghent University

SECTION 4

- **Failure costs**: these costs occur when the results of work fail to reach quality standards.

1. Can you give us an estimation of your yearly **rework** costs? (i.e. the amount of direct labor and material required to correct nonconforming material)
   = salary of employees + product loss

   €____

2. Can you give us an estimation of your yearly **scrap** costs? (i.e. the amount of non -conform products you have to throw away)
   = salary of employees + product loss

   €____

3. Can you give us an estimation of your yearly **breakdown maintenance** costs? (i.e. the maintenance is carried out when machines are down completely)
   =cost of breakdown maintenance + loss of sales

   €____

4. Can you give us an estimation of your yearly **warranty costs**? (i.e. the costs of service of sold items returned by the costumer for nonconformance, and the expense of replacement, repair, or service)
   = costs of service (time) + costs of replacement/refund

   €____

5. Can you give us an estimation of your yearly **discounts** given to customers due to non-conformities costs?
   =cost of discounts

   €____

6. Can you give us an estimation of your yearly **recall** costs?
   =product disposition costs + lost future sales + food safety consultant fees + costs associated with extra cleaning and sanitation + costs of implementing corrective actions + lab fees + government, legal, other fees/fines + employee overtime.

   €____

© Ghent University

This is the end of the questionnaire. Thank you for your participation!

© Ghent University
